# Asymptomatic syringomyelia accompanied with metastatic cerebellar and spinal intramedullary lymphoma: A case report

**DOI:** 10.1097/MD.0000000000008593

**Published:** 2017-11-17

**Authors:** Jia-Jia Zhou, Jin-Feng Xu, Xu-Ning Zheng, Guo-Ping Peng

**Affiliations:** a Department of Neurology; b Department of Radiology, First Affiliated Hospital, Zhejiang University School of Medicine, Hangzhou, China.

**Keywords:** asympotamic syringomyelia, diagnosis and management, metastatic lymphoma, spinal cord

## Abstract

**Rationale:**

Asympotamic syringomyelia accompanied with metastatic cerebellar and thoracic spinal intramedullary lymphoma is rare in clinical practice. If the intramedullary lymphoma is large enough, the patient will rapidly develop neurologic signs of spinal injury. The prognosis of this type of complication is always bad.

**Patient concerns:**

Rapid and correct diagnosis and treatment is important for metastatic extranodal lymphoma with B cell of origin.

**Diagnoses:**

Syringomyelia accompanied with metastatic cerebellar and thoracic spinal intramedullary lymphoma.

**Interventions:**

The patient was treated with a combination of systemic chemotherapy and focal radiotherapy and intrathecal therapy.

**Outcomes:**

Resolution of metastatic lymphoma was not continued after conservative medical management and the patient died finally due to multiple organ failure.

**Lessons:**

Syringomyelia can develop due to the metastatic thoracic intramedullary lymphoma in patients with diffuse malignant large B cell lymphoma. Early and accurate diagnosis, anti-lymphoma treatment, and timely neurosurgical intervention may delay the development of the disease.

## 1 Introduction

Diffuse large B-cell lymphoma (DLBCL) is a common type of malignant lymphoma, and DLBCL in the central nervous system (CNS) is an infrequent event with fatal consequences usually.^[1]^ Syringomyelia is a progressive anomaly characterized by a formation of an intramedullary cavity, and segmental dissociated sensory dysfunction is always the main clinical sign, though sometimes is asymptomatic.^[2]^ Syringomyelia can be caused by the tumor mass nearby, or are sometimes idiopathic.^[3]^ In this case, we report an elderly patient has asymptomatic syringomyelia that is accompanied with the metastatic cerebellar and thoracic spinal intramedullary lymphomas, which is rare clinically.

## 2 Patient's presentation

**Figure 1:**
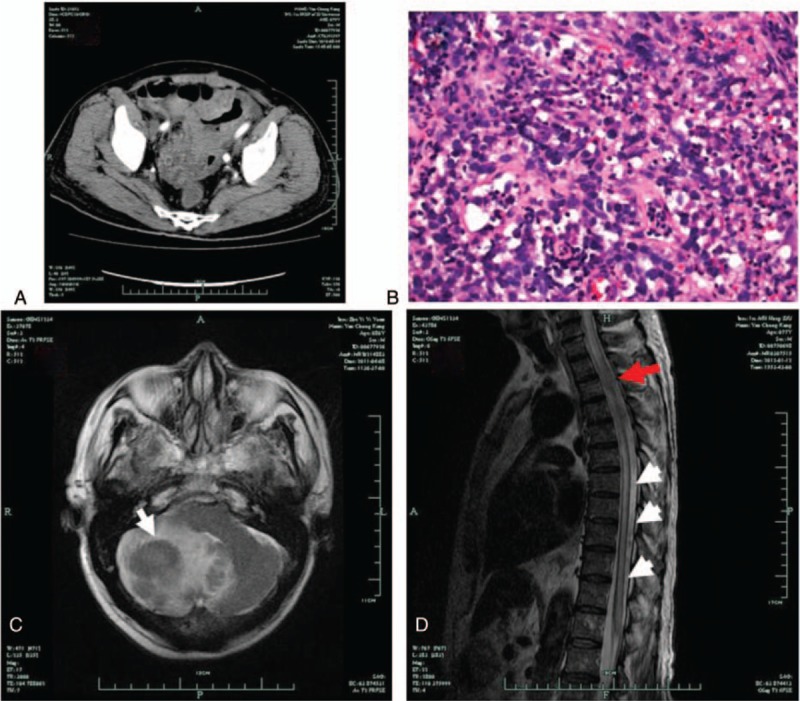
(A) Abdominal CT shows an ileal thickening with multiple mesenteric and retroperitoneal lymph nodes. (B) The pathological staining of endoscopic biopsy specimen shows a high cellularity, consisted of small tumor cells with interspersed islands of cells with neuronal differentiation (HE ×40). (C) Cranial MRI (with gadolinium enhancement) shows several atypic mass located in the cerebellar hemispheres with peripheral edema. (D) Thoracolumbar spinal MRI shows enlargement of the syrinx cavity (white arrow) which demonstrates the same signal intensity as the CSF on T2-weighted imaging, and also shows (red arrow) a diffused and abnormal enhanced mass that was equal intense on T2W imaging. CT = computed tomography, CSF = cerebral spinal fluid, MRI = magnetic resonance imaging.

About 2 years ago, a 79-year-old retired man was admitted to the hospital because of dull abdominal pain with nausea, constipation, and a poor appetite which lasted more than a week. His general condition, physical examination, and medical history were uneventful. Laboratory assay, chest x-ray, and electrocardiogram were normal. Abdominal enhanced computed tomography (CT) showed ileal thickening with multiple mesenteric and retroperitoneal lymph nodes, which indicated ileocecal lymphoma (Fig. 1A). The pathological staining using hematoxylin-eosin (HE) for the colonoscopy biopsy specimen got from the ileocecal region showed a high cellularity, consisted of small tumor cells with interspersed islands of cells with neuronal differentiation (Fig. 1B), and the further immunohistochemistry staining suggested a diffuse large B-type of non-Hodgkin lymphoma (CD20+, CD45+, and CD3−). The patient then received ileocecal tumor resection and the tissue pathological staining confirmed the colonoscopy biopsy results. After postoperative recovery, the patient received a systemic chemotherapy with cyclophosphamide, doxorubicin, vincristine, and prednisone (CHOP) plan for 13 cycles.

A year after this treatment, the patient developed dizziness and unsteady walking suddenly, accompanied with slight headache especially apparent when he moved his head abruptly. The patient was thus readmitted and general physical examination was normal. Neurological examination showed slight right-side hemiataxia and a positive Romberg test. A brain magnetic resonance (MR) imaging showed several masses in the cerebellar hemispheres with peripheral edema, suggesting metastatic lymphoma (Fig. 1C). The following cervical MR imaging showed no abnormal findings. The patient was then received Mannitol dehydrate therapy and another 6 cycles of systemic chemotherapy. When his condition was stabilized, he was transferred to a rehabilitation center whereby he underwent several courses of local radiotherapy for the brain lesions. He recovered partially afterwards, and his general condition improved.

Six months following the treatment, the patient felt fatigue and weakness of his lower extremities, and he could not get out of bed or walk. Neurological examination revealed 2/5 of normal muscle strength in both his lower extremities. Deep tendon reflexes were normal in upper extremities and enhanced in lower extremities. Babinski signs were presented bilaterally. There was no evidence of sensory disturbances or motor weaknesses in the upper trunk and upper extremities. Lumbar MR imaging showed lumbar degeneration and a L5/S1 slightly herniated disk (not shown). However, enhanced thoracic MR imaging revealed an intramedullary lesion of T2–3 spinal cord level with abnormal enhancement and thoracolumbar syringomyelia (Fig. 1D). A presumptive diagnosis of metastatic spinal intramedullary malignant lymphoma was proposed, however, the patient refused further examination including pathological biopsy. He was then treated with spinal cord radiotherapy and systemic chemotherapy with intrathecal injections of methotrexate. However, the patient died of multiple organ failure 3 months later as his condition worsened. Due to lack of consent from family members of the patient, autopsy was not conducted.

## 3 Discussion

Diffuse large B-cell lymphoma is a common type of malignant lymphoma, and the ascending colon and rectum more commonly affected.^[1]^ In this case, the patient was originally diagnosed as primary colorectal DLBCL, and received ileocecal tumor resection and secondary chemotherapy. Following the progression of lymphoma, cerebellum and thoracic spinal cord were then affected. Secondary CNS lymphoma was related to the direct invasion of lymphoma cells to CNS or spread to the brain and spinal along the subarachnoid cavity by the cerebrospinal fluid. The outcome of DLBCL with secondary CNS involvement was poor. Factors that were found to increase the risk of CNS involvement were advanced stage, high age-adjusted International Prognostic Index, bone marrow involvement, elevated lactate dehydrogenase level, involvement of more than 1 extranodal site, and testicular involvement.^[4]^ CNS involvement by aggressive lymphoma is an extremely heterogeneous and very complex situation, with many variables determining treatment of choice and outcome, including the B-cell-of-origin subtype. Comprehensive treatment including intrathecal therapy, head radiotherapy, and systemic chemotherapy are recommended. Intensification chemotherapy and autologous hematopoietic stem cell transplantation might also be helpful.^[5]^

The pathogenesis and natural history of syringomyelia is often related to trauma, tumor, and congenital abnormalities, but can be idiopathic as well.^[6]^ It is generally accepted that the presence of a syringomyelia with Chiari type I malformation is a strong indication for surgical decompression as the syrinx will progress, even if it is asymptomatic.^[7]^ In this case, the lesion located at the T2–3 level on spinal MR was the cause of the patient's leg weakness. Moreover, the syringomyelia located in the thoracolumbar cord was exposed together with the metastatic lymphoma. The asymptomatic syringomyelia may have developed due to the metastatic lymphoma since there was no abnormal finding on the cervical MR scan 6 months before. However, it supposed to be too early for a syrinx to develop with the appearance of metastatic lesion. Landan et al,^[8]^ have reported a case of syringomyelia secondary to primary CNS lymphoma and assume that the possible reason was the necrosis of centrally located tumor with liquefaction followed by absorption of necrotic tissue. And similarly, we suppose that maybe the same cause of asymptomatic syringomyelia in our case, however, the real reason for the development of syringomyelia is still difficult to ascertain.

A literature search revealed no prior case report of metastatic cerebellar and spinal lymphoma that accompanied thoracolumbar syringomyelia. The aim of this case report was not to describe a new association of diseases, but to alert practitioners to the possibility of association between these 2 neurological diseases. We emphasize the importance of establishing early diagnosis in patients with neurological symptoms, in order to establish therapeutic management which will improve the quality of life for these patients.

In all, we report a case with an asympotamic syringomyelia that accompanied metastatic cerebellar and thoracic spinal intramedullary lymphoma, which are rare in clinical practice. We illustrate the difficulty in diagnosis and therapeutic management and suggest the physicians to pay attention to such rare conditions.

## References

[1] Bjorkholm M Hagberg H Holte H Central nervous system occurrence in elderly patients with aggressive lymphoma and a long-term follow-up. Ann Oncol 2007;18:1085–9. 17363838 10.1093/annonc/mdm073

[2] Terae S Hida K Sasaki H Diagnosis of syringomyelia and its classification on the basis of symptoms, radiological appearance, and causative disorders. Brain Nerve 2011;63:969–77. 21878699

[3] Wang CC Adult medulloblastoma associated with syringomyelia: a case report. Cancer Biol Med 2012;9:137–40. 23691470 10.3969/j.issn.2095-3941.2012.02.011 PMC3643653

[4] Zahid MF Khan N Hashmi SK Central nervous system prophylaxis in diffuse large B cell lymphoma. Eur J Haematol 2016;97:108–20. 27096423 10.1111/ejh.12763

[5] Peñalver FJ Sancho JM de la Fuente A Guidelines for diagnosis, prevention and management of central nervous system involvement in diffuse large B-cell lymphoma patients by the Spanish Lymphoma Group (GELTAMO). Haematologica 2017;102:235–45. 27846613 10.3324/haematol.2016.149120 PMC5286932

[6] Velizarova R Crespel A Juntas-Morales R Teaching neuroImages: benediction sign as a result of cervical astrocytoma with syringomyelia. Neurology 2011;77:e50. 21876192 10.1212/WNL.0b013e31822c619d

[7] Marin SA Skinner CR Da Silva VF Posterior fossa arachnoid cyst associated with Chiari I and syringomyelia. Can J Neurol Sci 2010;37:273–5. 20437943 10.1017/s0317167100010064

[8] Landan I Gilroy J Wolfe DE Syringomyelia affecting the entire spinal cord secondary to primary spinal intramedullary central nervous system lymphoma. J Neurol Neurosurg Psychiatry 1987;50:1533–5. 3694211 10.1136/jnnp.50.11.1533 PMC1032570

